# Relationship between hemolysis and lipid oxidation in red blood cell-spiked fish muscle; dependance on pH and blood plasma

**DOI:** 10.1038/s41598-024-52090-8

**Published:** 2024-01-23

**Authors:** Semhar Ghirmai, Annika Krona, Haizhou Wu, James Whalin, Michael Axelsson, Ingrid Undeland

**Affiliations:** 1 Division of Food and Nutrition Science, Department of Life Sciences, Chalmers University of Technology, 412 96 Gothenburg, Sweden; 2 Division Bioeconomy and Health, Department Agriculture and Food, RISE Research Institutes of Sweden, Frans Perssons Väg 6, 402 29 Gothenburg, Sweden; 3 Department of Biological and Environmental Sciences, Gothenburg University, Medicinaregatan 18a, 413 90 Gothenburg, Sweden

**Keywords:** Lipids, Biochemistry

## Abstract

The relationship between hemolysis and lipid oxidation was explored in red blood cell (RBCs)-spiked washed cod mince (WCM). At pH 6.8 and 3 ± 1 °C, intact RBCs (71 µM Hb) delayed lipid oxidation by 1 day compared to WCM with partly or fully lysed RBCs which oxidized immediately. Intact RBCs also lowered peak peroxide value (PV) and thiobarbituric acid reactive substances (TBARS) with up to 59.5% and 48.1%, respectively. Adding 3% (v/w) blood plasma to RBC-spiked WCM delayed the lipid oxidation onset from 1 to 3–4 days without delaying hemolysis. At pH 6.4 the oxidation onset in RBC-WCM was the same as for pH 6.8 while at pH 7.2–7.6 lipid oxidation was suppressed for 7 days. Micrographs revealed RBC-lysis from day 2 at pH 6.4 but at pH 7.6, RBC stayed intact for ≥ 7 days. Thus, assuring presence of plasma-derived antioxidants and/or elevating muscle pH to avoid hemolysis can aid valorization of blood rich underutilized fish raw materials.

## Introduction

Vast efforts are being made to achieve sustainable production and consumption of fish products. Industries and researchers are together working towards new routes to build circular blue bioeconomies through improved utilization of captured and harvested fish raw materials for food. A factor which however often hampers such developments is the high susceptibility of many poorly used fish raw materials towards lipid oxidation. This chemical reaction limits shelf-life through production of rancid odor and flavour, change in color and texture as well as reduced nutritional value^1^. Small pelagic fish species such as herring, mackerel, and sardine, just to mention a few, are especially prone to lipid oxidation due to their high content of hemoglobin (Hb), which is a strong lipid pro-oxidant^2,3^. The same applies to the blood rich side-streams emerging in fish filleting. It has earlier been suggested that Hb accounts for all the lipid oxidation capacity of blood^4^, since equal rancidity developed in washed cod mince (WCM) fortified with 5.8 µM Hb in the form of hemolysate or whole blood.

The main pathway by which Hb react with lipids is through cleavage of pre-formed lipid hydroperoxides (LOOH) by metHb, ferrylHb or hemin, which generate lipid radicals that can further propagate lipid oxidation^5,6^. Another pathway goes via formation of ferrylHb radicals which can attack intact fatty acids and thus act as initiators^5^. The last decades, researchers have studied several strategies by which Hb-mediated lipid oxidation can be reduced in fish raw materials or products, most of these being based on e.g. dipping, glazing, spraying or direct additions of different antioxidants or antioxidant-rich plant extracts^1^. We have lately explored a potential novel strategy to prevent lipid oxidation via limiting lysis of red blood cells (RBCs) and thereby reducing Hb contamination of the fish muscle^7,8^. This strategy originates in earlier observations showing that as long as the Hb is contained within the RBCs, it does not possess the same oxidative capacity as free Hb^4,9^.

In whole blood (WB) or washed and resuspended RBCs (wr-RBCs), we observed that trout and herring RBCs rapidly lyse in response to osmotic imbalance, temperatures > 6 °C and mechanic stress^7^. During processing, fish could be subjected to all these conditions, not least when integrating sea or tap water into the production line for storage or rinsing purposes, or when pumping fish though narrow sectors. Furthermore, we found that pH’s below neutrality, which is the case in *post mortem* fish muscle, increased hemolysis^8^ and stimulated oxidation of the RBC membrane while pH > 7 had the opposite effect. The latter was ascribed the pronounced Root and Bohr effects of fish Hbs along with the strong pro-oxidative properties of metHb and released hemin towards muscle lipids^5,6,10,11^.

In all our earlier trials, we found that RBCs always were more stable towards lysis in presence of blood plasma^7,8^. Blood plasma contains several components such as antioxidants, osmolytes and glucose that could be important for the RBC integrity^7,8^. Trout blood plasma was also previously shown to decrease Hb-mediated lipid oxidation in a WCM system^4^.

Beyond the mentioned studies of Richards and Hultin^4^ and Perez, Tatiyaborworntham^9^, no papers have been published where lysis of RBCs in a muscle matrix is linked to the development of lipid oxidation. Such studies would be of high importance to confirm whether the prevention of RBC lysis can be a new route to delay lipid oxidation in fish. The current study was conducted to expand our knowledge on: (1) the relationship between hemolysis and fish lipid oxidation and (2), the stability of RBC-spiked fish muscle towards hemolysis and lipid oxidation under different pH-conditions or presence of plasma. Experiments were carried out using trout RBCs and a fish model system consisting of washed cod mince (WCM).

## Material and methods

### Fish supply

Rainbow trout *(Oncorhynchus mykiss)* was obtained from Vännåns fiskodling AB, Sweden. The fish was maintained in tanks with aerated freshwater, ~ 10 °C, in the animal facility at Gothenburg University, Department of Biological and Environmental Sciences, Medicinaregatan 18. The fish was kept under a 12:12 photoperiod and fed commercially available trout pellets three times per week.

### Bleeding procedure

The terminal bleeding of trout was performed after the fish was killed by a blow to the head. Heparinized syringes and needles were then used to withdraw ~ 10 mL blood from the caudal vein, the blood was then stored on ice until further use within 24 h. The bleeding was performed in compliance with the PREPARE guidelines and the reporting follows the recommendations in the ARRIVE guidelines. The used procedures were also approved by the Gothenburg regional ethical committee, ethical permit number 5.8.18-06591-2019.

### Preparation of washed intact or lysed red blood cells

Hematocrit (hct) % of WB was measured according to Ghirmai, Eriksson^7^. The heparinized blood was centrifuged at 700 × g for 10 min at 4 °C to recover the plasma before proceeding with washing of the RBCs according to a modified protocol of Fyhn, Fyhn^12^. The first two washing steps of RBCs were performed in 0.9% NaCl containing 1 mM Tris–HCl pH 8.0, and the third washing was performed in 0.9% NaCl without adjusted pH to remove residual buffer trapped between the RBCs. Lysis of RBCs was prepared with 1 mM Tris–HCl pH 8.0 for 1 h on ice (Fyhn, Fyhn^12^. This method was also used to determine the total Hb of each washed concentrated RBC batch used in the study.

### Analysis of Hb-concentration

Hb concentration in RBCs, hemolysates and supernatants from the WCM model system (see below) was analyzed with Drabkin’s (cyanmetHb) method^13^. In each well of a 96-well microplate, 50 µL Drabkin’s solution was mixed with 50 µL RBCs/hemolysate/supernatant and the absorbance was measured at 540 nm on a microplate reader (Safire2; Tecan Group Ltd.). A standard curve was prepared using Hb from lyophilized bovine blood (Sigma-Aldrich, St. Louis, MO).

### Washed cod mince (WCM) model system

Fresh cod *(Gadus morhua)* fillet without skin (< 24 h post-mortem) was purchased from a local fish market (Landala Fisk, Gothenburg), and transported to the laboratory on ice. Dark muscle, blood spots and connective tissue was removed manually. The white muscle was cut into small pieces and minced using a kitchen-aid mixer (Ultra Power, Model KSM90, Kitchen aid, St. Joseph, Michigan USA). The mince was then washed in 3 volumes (w/w) cold distilled water, stirred manually for 2 min, and let to leach for 15 min at 4 ºC. The washed mince was filtered through a sieve and the steps were repeated a second time with 3 volumes (w/w) cold 50 mM phosphate buffer (PB) at pH 6.4, 6.8, 7.2 or 7.6 with the osmolarity adjusted by NaCl to reach final 308 mOsm/L. In the third wash, after addition of 3 volumes (w/w) PB, the mixture was homogenized with a polytron (Ultra Turrax, IKA Werks, Intermed Labassco) for 1 min 15 s at 15,000 rpm, and then let to leach for 15 min at 4 °C. The mince-washing solution homogenate was then centrifuged (Sorvall® Superspeed RC-5C Plus, Kendro Laboratory Products, Stockholm, Sweden) at 15,000 × g for 25 min at 4 °C. The supernatant was poured off and the remaining WCM was manually mixed to obtain a homogenous sample. The WCM was distributed into small zip-lock bags and stored in – 80 °C until further use. All steps, washing, de-watering and centrifugation were performed at 4 °C. Moisture content of the WCM´s was measured gravimetrically by subjecting ≥ 3 g of the samples to 105 °C for 24 h.

### Addition of red blood cells to WCM

Herring filleting side streams have previously been proven to contain 4.9 up to100 µmol Hb/kg mince depending on the exact part^14^. To create a realistic WCM model system, mimicking heme-rich fish muscle, a Hb concentration of 71 µM was therefore desired. In the first experiment, this amount of Hb was added in the form of fully lysed, fully intact, or partly lysed/partly intact RBCs. For the latter, 71 µM Hb was added as 25% lysed + 75% intact RBCs, 50% lysed/50% intact RBCs and 75% lysed/25% intact RBCs. These samples are hereafter denoted according to the % of the total Hb which was added as intact or lysed RBCs. To facilitate an even spreading of RBCs into the sample, the WCM was first diluted twofold with 50 mM PB of the desired pH. Based on the analyses of Hb concentration of washed RBCs or hemolysates (see above), the desired volume of concentrated RBCs or lysed RBCs was then carefully stirred into the WCM with a small stainless-steel spoon. Prior to addition of the pro-oxidant, 200 ppm of the antimicrobial agent streptomycin was added and manually mixed into the sample to limit bacterial spoilage.

To ensure uniform oxidation of the WCM sample and to avoid an oxygen gradient, the RBC-spiked WCM was then stored at 3 ± 1 °C in small petri-dishes (50 mm diameter) with maximum sample height of 4 mm, to allow oxygen penetration.

### Quantifying degree of hemolysis during storage of samples

**Figure 1:**
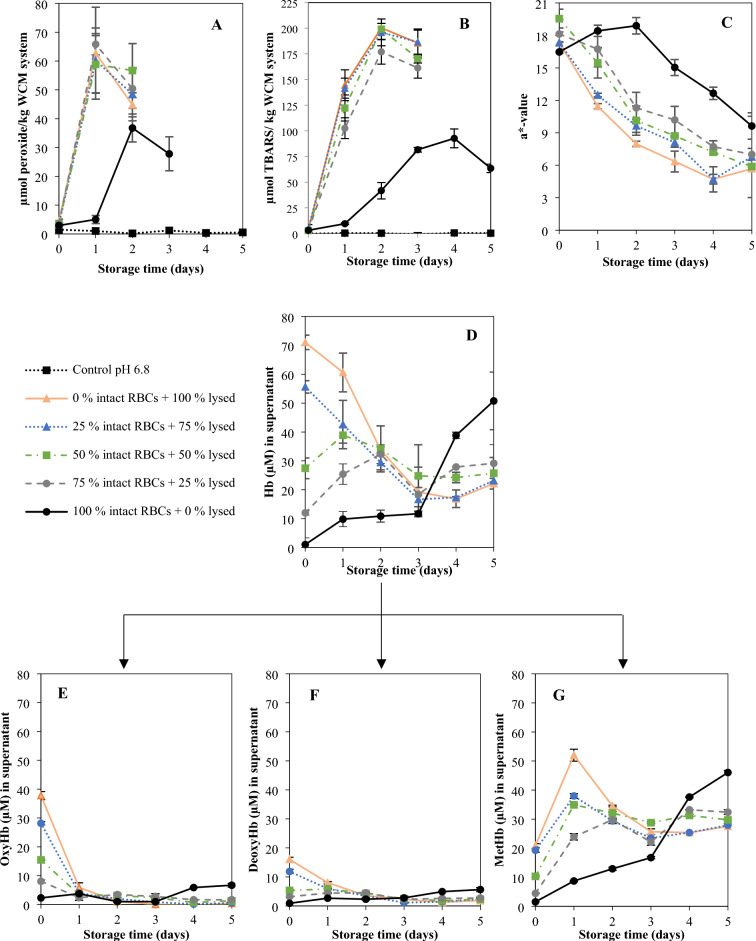
Lipid oxidation profile, PV and TBARS, of the RBC-spiked WCM system is displayed in (panel **A** and **B**), respectively, and (panel **C**) show the redness value (a*) of the WCM system. Data for total Hb (panel **D**) and the different Hb forms measured in the supernatant of the WCM system is displayed in (panel **E**) (oxyHb), (panel **F**) (deoxyHb) and (panel **G**) (metHb). The total concentration of Hb was 71 µmol/kg WCM.

**Figure 2:**
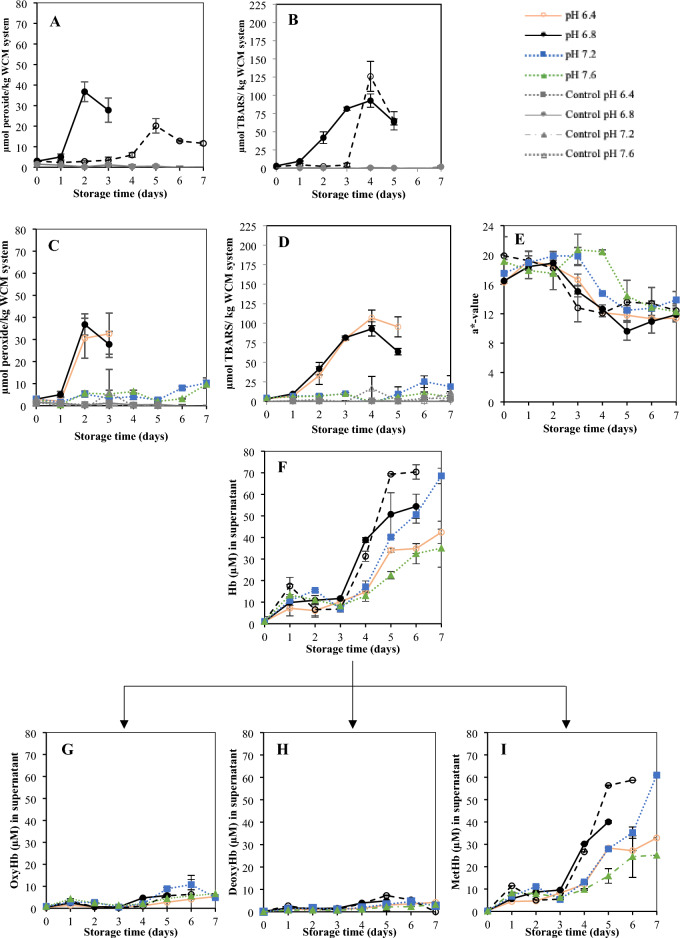
PV and TBARS in the RBC-spiked WCM system with and without plasma (panel **A** and **B**, respectively). PV and TBARS comparing the effect of various pH (panel **C** and **D**, respectively) as well as redness-change of the WCM system (panel **C**). Hb data, (panel **D**–**E**), are measured on the supernatant, (panel **D**) display total Hb and (panel **E**, **F**, and **G**) display oxy-, deoxy-, and metHb respectively. The total concentration of Hb was 71 µmol/kg WCM.

*Analyses of Hb in sample supernatants:* A 2g subsample of the RBC- or hemolysate-spiked WCM taken at start or during storage was centrifuged at 2000 × g for 4 min at 4 °C. The supernatant was transferred into a new tube and centrifuged at 7700 × g for 3 min at 4 °C for removal of small muscle fibers that did not sediment in the first centrifugation. Hb was then quantified according to Drabkin’s (cyanmetHb) method^13^ according to above. The data was then described as absolute quantities in the results and relative quantities are presented in the Supplementary (Fig. 1–2).

*Microscopic visualization of RBCs:* A droplet from the liquid phase of the WCM system was smeared on a microscope glass and left to dry overnight. After drying the cells were fixed in 4% paraformaldehyde in PBS for 10 min and rinsed in water before staining with hematoxylin and eosin. DNA in the nucleus is hereby stained blue and proteins in the cytoplasm, stained pink. The stained samples were examined in duplicates using an Olympus BX53 microscope (Olympus Life Science, Tokyo, Japan) and micrographs were captured with a CMos SC50 camera (Olympus Life Science) and processed with the Olympus software cellSense Entry.

### Estimation of oxy-, deoxy- and met-Hb in the supernatant of the WCM model

The recovered supernatant from the two centrifugation steps described in the previous section was also subjected to scanning in the wavelength range 700–500 nm to estimate the relative amounts of oxy-, deoxy- and metHb according to the Benesch equations^15^. The absorbance values at 630 nm, 576 nm and 560 nm were used for this purpose.

### Analyses of redness-loss during storage of WCM

Redness (a*) of the model system was measured with a colorimeter (Minolta Chroma Meter CR-300, Minolta Corp., Ramsey, NJ) on the bottom of the petri-dish without stirring. Thus, RBCs sediment to the bottom over time. Calibration was performed with a white Minolta calibration plate with a D_65_ illuminant and 2° observer. To perform the measurement, a probe was pressed against the bottom flat surface of the petri dish at 5 different locations of the flat surface. The five readings were then used to get an average value of the sample.

### Analysis of lipid oxidation during storage of WCM

Subsamples of 2 g were extracted as described by Undeland, Hultin^16^ using 20 mL chloroform: methanol (1:1) containing 0.05% w/v butylhydroxytoluene (BHT). Peroxide value (PV) was analyzed in the chloroform phase recovered from the extraction using the ferric thiocyanate method^16^. Thiobarbituric acid reactive substances (TBARS) were analyzed in the water–methanol phase^17^.

### Expression of results and statistical evaluations

Storage experiments were performed in duplicates (n = 2) with two technical replicates (r = 2). The results are expressed as mean values from the two experiments ± standard deviation (SD). Lipid oxidation data are visualized only until the peak values started to decline, since the main interests were to record the height of the peak and the length of the lag-phase rather than the subsequent interactions between hydroperoxides and carbonyls with e.g., proteins. Experimental results were statistically analyzed with analysis of variance (ANOVA), with time, treatment and replicate treated as nominal variables and the response variable, e.g., TBARS treated as a continuous variable. When significant effect of treatment was observed, a post-hoc Tukey test was used to determine differences between treatments. Time*treatment interactions were observed, but interactions did not decrease significance of treatment effects over time, and so were not reported for each time*treatment occurrence. The response variable (e.g., PV, TBARS) normality was analyzed with a residual plot, and were normalized with a log_10_ transformation as needed. Significance was set at *p* < 0.05 for all experiments. JMP Pro version 17.0.0 (JMP Statistical Discovery LLC) was used to statistically analyze data. Correlation analyses were performed by calculation of Spearman’s correlation coefficient in Microsoft Excel, for those responses that showed monotonical relationship.

## Result and discussion

Recent studies have revealed that lipid oxidation starts within 1 day on ice in minced herring fillets and in sorted minced side-streams from the filleting operation (heads, backbones, belly flap plus intestines and tails), although with different development rates^14^. One of the main factors correlating significantly to oxidation rate was the Hb-level in the five different herring cuts studied. In this study, the hypothesis that Hb-mediated fish lipid oxidation can be mitigated by maintaining RBC integrity was examined in a fish muscle model system to bring our previous findings from RBC model systems^7,8^ closer to a fish in situ scenario.

### Lipid oxidation, soluble Hb-levels and Hb-forms in WCM with different ratios of intact RBC

To study how the degree of RBCs integrity affect the formation of lipid oxidation products in a fish model system, WCM (pH 6.8) was spiked with different ratios of lysed and/or intact RBCs. WCM was selected as model since it has the structure of muscle with intact myofibrils and cellular membranes but lacks most endogenous pro- and antioxidants thereby allowing the effect of added oxidants to be studied separately^18,19^.

**Figure 3:**
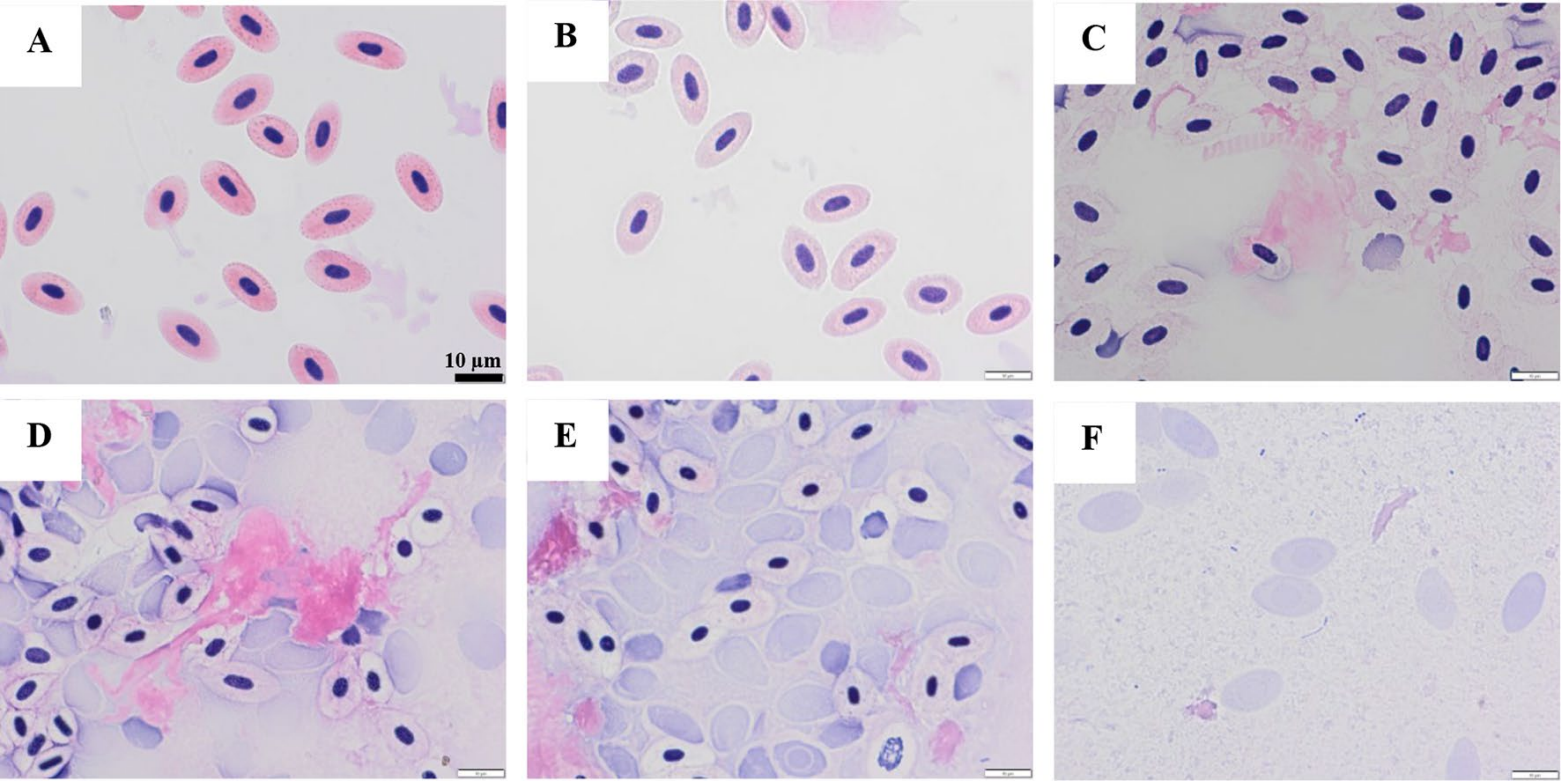
Micrographs of RBCs from the aqueous phase of the WCM system at pH 6.4 following staining with eosin and hematoxylin, day 0 (panel **A**), day 1 (panel **B**), day 2 (panel **C**), day 3 (panel **D**), day 4 (panel **E**), day 7 (panel **F**). Scale bar 10µm.

The control sample at pH 6.8, which had no Hb added (i.e., either intact or lysed RBCs), did not develop any lipid oxidation over the 5 days at 3 ± 1 °C as displayed in Fig. 1A,B. Samples with Hb added as 0–75% intact RBCs plus 100–25% lysed RBCs displayed rapid PV and TBARS development from the first day of storage. The lipid oxidation profiles of these samples were not significantly different; thus, the 17.8 µM free Hb emerging from the 25% lysed RBCs was enough to yield as fast and intense lipid oxidation as fully lysed RBCs (i.e., 71 µM free Hb). However, given the very short oxidation lag phase, it could not be ruled out that there were significant differences between samples during the first day of storage. An extra trial with the WCM-samples containing either 100% lysed or 50% lysed/50% intact RBCs was therefore carried out to monitor oxidation more frequently in the time span of 1–2 days (Supplementary, Fig. 3). Results revealed that the sample with 100% lysed RBCs did not provide a shorter lag phase or significantly (*p* < 0.05) higher oxidation product level compared to the sample with 50% intact RBCs. This suggests that with the extensive amount of Hb used in these trials (71 µM), the membrane lipid substrates of the WCM and RBCs rather than the Hb-level may become the limiting factor for maximum oxidation product levels reached, contradicting our earlier studies done at lower Hb-levels^16^.

A 1-day delay in the onset of lipid oxidation was provided by maintaining RBCs 100% intact from start. Complete RBC integrity also significantly (*p* < 0.05) lowered maximum PV and TBARS values by 59.5 ± 2.9% and 48.1 ± 2.9%, respectively (Fig. 1A,B). These data agreed with the findings earlier reported by Richards and Hultin^4^ revealing 1 day longer oxidation lag-phase when ice-storing WCM (pH 6.3) with 5.8 µM Hb added as intact instead of lysed RBCs. The same study showed that only 0.5 µM Hb in the form of hemolysate was needed to initiate lipid oxidation under the described conditions, revealing that the 71 µM Hb chosen here to mimic blood-rich fish filleting side streams^1,14^ were far above Hb-levels required to initiate lipid oxidation in WCM. In the sample with 100% intact RBCs, a maximum of 1.30 ± 0.14 µmol MDA equivalents were reached per mole Hb tetramer added, which was lower compared to samples with partly or fully lysed RBCs (2.49–2.82 ± 0.14 MDA equiv./Hb tetramer) (Fig. 1B), indicating that less Hb was available for oxidation reactions. Across several earlier studies performed in WCM at different pH´s, strikingly constant ratios between maximum TBARS and added Hb have been described (6.7–24 µmol MDA equivalents per mole Hb tetramer, n = 12)^16^ demonstrating that Hb act as a reactant rather than a catalyst. That the ratios in our study were lower than earlier reported ones could be due to the sedimentation of intact RBCs in the WCM; causing a RBC-gradient which restricted released Hb to react with lipids in the nearby bottom microenvironment. In this case, it is possible that the lipid substrate became limiting for the maximum amount of oxidation products formed. Along the same lines, we hypothesize that the absence of difference between the maximum TBARS-to-Hb-ratios in WCM fortified with *fully* lysed RBCs (i.e., without an observed gradient) vs. with 25–75% lysed RBCs, is explained by the excessive amount of Hb available, rendering the lipid substrate limiting for TBARS-levels formed.

Redness of the WCM system (Fig. 1C) as well as Hb-level (Fig. 1D) and Hb-forms in the supernatant (Fig. 1E–G), were analyzed to relate the development of lipid oxidation with storage-induced hemolysis and Hb autoxidation. An initial increase in redness (a*) value (Fig. 1C) was observed in the sample spiked with fully intact RBCs. This increase was most likely explained by the visually observed sedimentation of the RBCs to the bottom of the petri-dish; which is the site where the color was measured. The same phenomenon was not found for samples containing 25–100% lysed RBCs as free Hb did not sediment throughout the storage. After two days, a rapid loss of redness was then observed in the sample with only intact RBCs, continuing to the end of the storage. In all other samples, i.e., with partially or fully lysed RBCs, redness decreased already from the start of the storage trial due to rapid autoxidation of oxyHb/deoxyHb to metHb and/or potential oxidative destruction of the heme-ring^20,21^. A fast transition from oxy-/deoxyHb to metHb was confirmed by analyses of the supernatant from WCM-samples with 25–100% lysed RBCs (Fig. 1E–G). Most Hb autoxidation took place within the first 1–2 days, after which metHb levels decreased. In the sample fortified with 100% intact RBCs, metHb formation in the supernatant was slow in the first 3 days followed by a sharp increase (Fig. 1G), which correlated to the decrease in redness after day 2 in this sample (Fig. 1C).

As can be seen in Fig. 1D, the quantities of total Hb in the supernatant of WCM spiked with 100% intact RBCs followed the same kinetics as the metHb-levels (Fig. 1G); i.e., a small increase during the first day, followed by a lag phase until day 3. Thereafter, a rapid increase in soluble Hb was found, pointing at fast lysis of the RBCs. Samples spiked with high ratio of lysed to intact RBCs (100/0 and 75/25) however showed a decreasing trend in soluble Hb over time, the same also being observed after 1 and 2 days in samples with 50/50 or 25/75 lysed to intact RBCs, respectively (Fig. 1D). A likely explanation was that metHb (Fig. 1G) or released hemin was either binding to the WCM or was degraded e.g., by reactive oxygen species (ROS) such as hydrogen peroxide (H_2_O_2_) and superoxide (O_2_^•−^) in the system^20,21^. In metHb, the hemin-group is up to 60-folds less anchored compared to oxy-form, thus, stimulating a fast release^2,3^. The lipophilic properties of hemin make the WCM a likely site for dissolving and/or binding given its high levels of cellular membranes and myofibrils with potential hydrophobic sites. It is for example described that the propionate group of hemin can bind through hydrophobic attraction or electrostatic interaction to amines of the phospholipid headgroup^22^. Overall, more soluble Hb could be recovered at the end of the storage in the supernatant from the sample fortified with 100% intact RBCs (72%), compared to samples spiked with 25–100% lysed RBCs (~ 35% Hb), confirming Hb-binding and/or destruction.

### Effect of pH and plasma on lipid oxidation, soluble Hb-levels, and Hb-forms in WCM spiked with intact RBCs

Trout WB hct % was estimated to ~ 39%, meaning that ~ 60% is plasma. This ratio between RBCs and plasma was maintained when adding these two components to the WCM system, yielding a final plasma concentration of only 3% (v/w). Still, plasma decreased PV and TBARS in presence of 100% intact RBCs (*p* < 0.05; Fig. 2A,B). The antioxidative capacity of plasma was in line with earlier findings of Richards and Hultin^4^ revealing that plasma added to WCM at 2.5% (w/w) together with hemolysate at 1.8 or 5.8 µmol Hb/kg WCM delayed the onset of lipid oxidation by 1 day compared to a plasma-free control. Plasma contains many different antioxidant molecules and enzymes acting through a variety of mechanisms^23^. For example, glutathione peroxidase and catalase can eliminate hydroperoxides without formation of lipid radicals^24^ while chain-breaking antioxidants such as tocopherol and the enzyme superoxide dismutase (SOD) can trap peroxyl and superoxide radicals, respectively^25^. The addition of plasma to the WCM did however not affect the release of Hb from RBCs to the supernatant (Fig. 2F) compared to a sample kept at the same pH (6.8) without plasma. That we could not repeat the hemolysis-delaying effect of plasma seen in a wr-RBC model system^7,8^ was most likely due to the high plasma levels used in that system (12–75% v/w), compared to the 3% (v/w ) in the WCM-system. An interesting finding was that plasma seemed to hinder Hb binding to the WCM. While 70.4 µM Hb (i.e., 99% of the added Hb) was measured in the supernatant at the end of the storage when plasma was present, only 54.4 µM Hb (77% of added Hb) could be recovered without plasma, after 6 days of storage. This could be explained by possible binding of Hb to proteins in blood plasma such as albumin^26^, haptoglobin (Hp)^27,28^ and hemopexin^29^ rather than to myofibrillar WCM proteins. However, it could also be linked to lower binding to membranes; in earlier studies of antioxidative properties of fish muscle press juice (PJ) -which also comprises plasma- PJ prevented the binding of Hb to sarcoplasmic reticulum (SR)^30^. Whether the antioxidative effect of plasma also involves less hemin-release remains to be proven.

In our previous paper^8^ we found that increasing the pH of the incubation solution for wr-RBCs from 6.4–6.8 to 7.2–8.0 decreased the rate of hemolysis and suppressed RBC membrane lipid oxidation. Similarly, increasing the pH to > 6.8, clearly decreased TBARS (*p* < 0.05) and increasing pH > 7.2 decreased lipid peroxides (*p* < 0.05) of the RBC-WCM system (Fig. 2C,D). At pH 7.2 and 7.6, PV and TBARS values kept below 10.3 µM and 25.3 µM, respectively, during the whole storage period, results which were in line with earlier studies of lipid oxidation in hemolysate-spiked WCM^31,32^. Samples with higher pH also had a more preserved redness (Fig. 2E), clearly re-confirming how metHb-formation and lipid oxidation go hand in hand (Wetterskog and Undeland 2004). The phase of fast redness-decrease appeared after 4 and 3 days in samples at pH 7.6 and 7.2, respectively, while it appeared in the other samples already after 1–2 days. In all samples, the redness-loss levelled off after 5 days (Fig. 2E).

**Figure 4:**
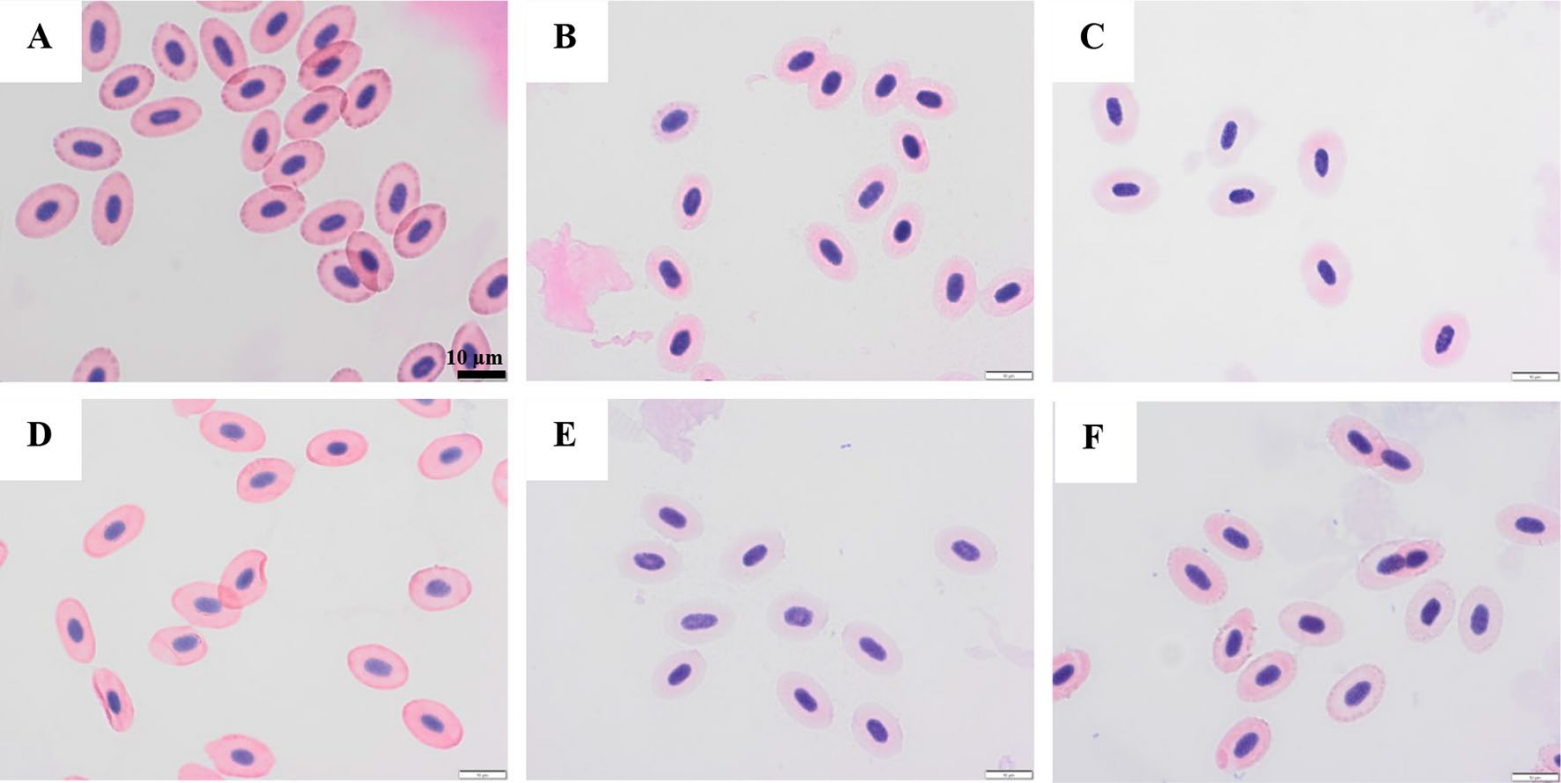
Micrographs of RBCs from the aqueous phase of the WCM system at pH 7.6, after staining with eosin and hematoxylin, day 0 (panel **A**), day 1 (panel **B**), day 2 (panel **C**), day 3 (panel **D**), day 4 (panel **E**), day 7 (panel F). Scale bar 10µm.

Figure 2F shows how the level of Hb in the supernatant was also dependent on pH. All samples (pH 6.4–7.6) displayed ~ 7.1 µM Hb in the supernatant already after 1 day, which means ≥ 10% of hemolysis. A plateau was then seen, and from day 3, the most rapid Hb release was measured in the two samples adjusted to pH 6.8. The total Hb in the supernatant of the sample at pH 6.8 without plasma levelled off at a value of 54.4 µM, which is 77% of the Hb-level added prior to storage. The pH 7.2-sample increased second fastest in soluble Hb-level and reached nearly the maximum 71 µM after 7 days. The sample adjusted to pH 6.4 showed a significantly reduced (*p* < 0.05) amount of soluble Hb at day 7 compared to samples at 6.8–7.2, with only 42 µM Hb recovered in the supernatant. This indicated that 41% Hb/hemin had bound to WCM, was degraded or was present as intact RBCs. The latter was however less likely based on our earlier studies of wr-RBCs^8^. The sample with pH 7.6 had the lowest amount of soluble Hb, 35 µM Hb, at day 7, which was hypothesized to be due to limited lysis. Microscopy was used as a complementary method to confirm the presence/absence of hemolysis in the pH 6.4/7.6-samples (see below and Figs. 3, 4). Examining Fig. 2C,D together with Fig. 2F thus reveals that in samples at pH 6.4 and 6.8, lipid oxidation started *before* the rapid release of Hb occurred from RBCs, i.e., when only 7.1 and 9.8 µM Hb, respectively, could be measured in the supernatant. This indicates that in blood-rich systems, even minimal hemolysis becomes critical for the lipid oxidation onset, which is in line with the earlier findings that only 0.5 µM Hb was needed to initiate lipid oxidation of WCM at pH 6.3^4^.

The increased pro-oxidative effect of released Hb at lower pH (6.4–6.8) can be explained by several mechanisms. One is a likely high concentration of the hydroperoxyl radical (•OOH) which can partition into membranes^33^. Second, hydrogen peroxide (H_2_O_2_) can be formed from superoxide anion radicals (•O_2_^−^)^34^ generated in the oxidation of oxyHb to metHb. H_2_O_2_ can oxidize oxy/deoxyHb into metHb, ferrylHb or ferrylHb radicals as well as generate hydroxyl radicals (•OH) according to the Fenton reaction with oxyHb^5,6,10,35^. A third reason can be that the relative oxygenation of trout Hb drastically decrease at pH below 7.0^31^ due to the Root effect, accelerating autoxidation of oxyHb to metHb, the latter which can propagate lipid oxidation e.g. via breakdown of lipid hydroperoxides^5,6,10,35^. Increased metHb formation can also accelerate loss of the hemin since it is more weakly anchored in met- compared oxy-Hb, as described above^2,3^; the hydrophobic nature of hemin stimulates its partitioning into membranes and thereby its proximity to e.g., lipid hydroperoxides^11^. In addition, HbIV, which is the most abundant and reactive Hb type in fish blood^36^ is anodic with an isoelectric point (pI) of 6.2–6.3^37^. Thus, at lower pH, there will be less electrostatic repulsions from the negatively charged membranes, facilitating Hb-membrane interactions. It has been described that such interactions stimulate the dissociation and liberation hemin from the Hb pocket into the membrane interior^38,39^. Thus, different modes of binding between Hb/heme and the WCM < pH 7.2 appear to be central both for the development of lipid oxidation and for the difficulties in following soluble Hb as a mode of monitoring hemolysis.

Analyses of the distribution between Hb-forms in the supernatant (Fig. 2G–I) revealed that Hb oxidized fast in all samples once it was released from the RBCs. This could be explained by e.g., the presence of effective metHb reducing enzymes such as NADH-dependent cytochrome b5 reductase and NADPH metHb reductase, inside trout RBCs^40^. Another explanation, could be the dissociation of the tetrameric Hb molecule into dimers upon dilution, which oxidize faster and readily lose its hemin, compared to the intact Hb-molecule^41^. Interestingly, at the end of the storage period, the samples adjusted to pH 7.2 and 7.6 contained as much as 61.0 and 25.1 µM metHb, respectively, in the supernatant, yet lipid oxidation was suppressed. It is hypothesized that at the higher pH´s, electrostatic repulsions between HbIV and the membrane reduces interactions between the two^38^ thereby mitigating hemin-dissociation into the hydrophobic membrane interior. Further trials are however needed to confirm this hypothesis.

Microscopy was used for the RBC-spiked WCM samples at pH 6.4 (Fig. 3) and 7.6 (Fig. 4) to follow their hemolysis over time and confirm the nature of the low Hb-levels of their supernatants. At day 0, for both pH’s, the RBCs show an elongated disc-shape with a compact nucleus stained in blue within a pink cytoplasm. This morphology revealed that RBCs were intact. Already after 1 day, minor bleaching of the cytoplasm could be seen in the sample with low pH (Fig. 3B), which could indicate Hb-diffusion via small pores in the membrane due to oxidation^42,43^. After 2 days of storage, drastic morphological deformation was observed with nucleic content filling up the whole RBC (Fig. 3C). At day 7, no intact RBCs could be found in this sample (Fig. 3F). At pH 7.6, deformed RBCs or leakage of the nucleic content inside the RBC could not be observed throughout the entire storage, revealing that RBCs stayed intact (Fig. 4). Combining the information from Hb measured in the supernatant (Fig. 2F) and the micrographs (Figs. 3, 4) it can thus be concluded that all RBCs were lysed at pH 6.4 within the storage time of 7-days, although only 59% of the total Hb could be detected in the supernatant, confirming Hb-binding or degradation at low pH.

### Correlation between redness, lipid oxidation, soluble Hb or Hb-forms

Figure 1 PV an d TBARS in the RBC-spiked WCM system (panel A and B, respectively) as well as redness-change of the WCM system (panel C). Hb data, panel D-E, are measured on the supernatant, panel D display total Hb and panel E, F, and G display oxy-, deoxy-, and metHb respectively. The total concentration of Hb was 71 µmol/kg WCM.

**Figure 5:**
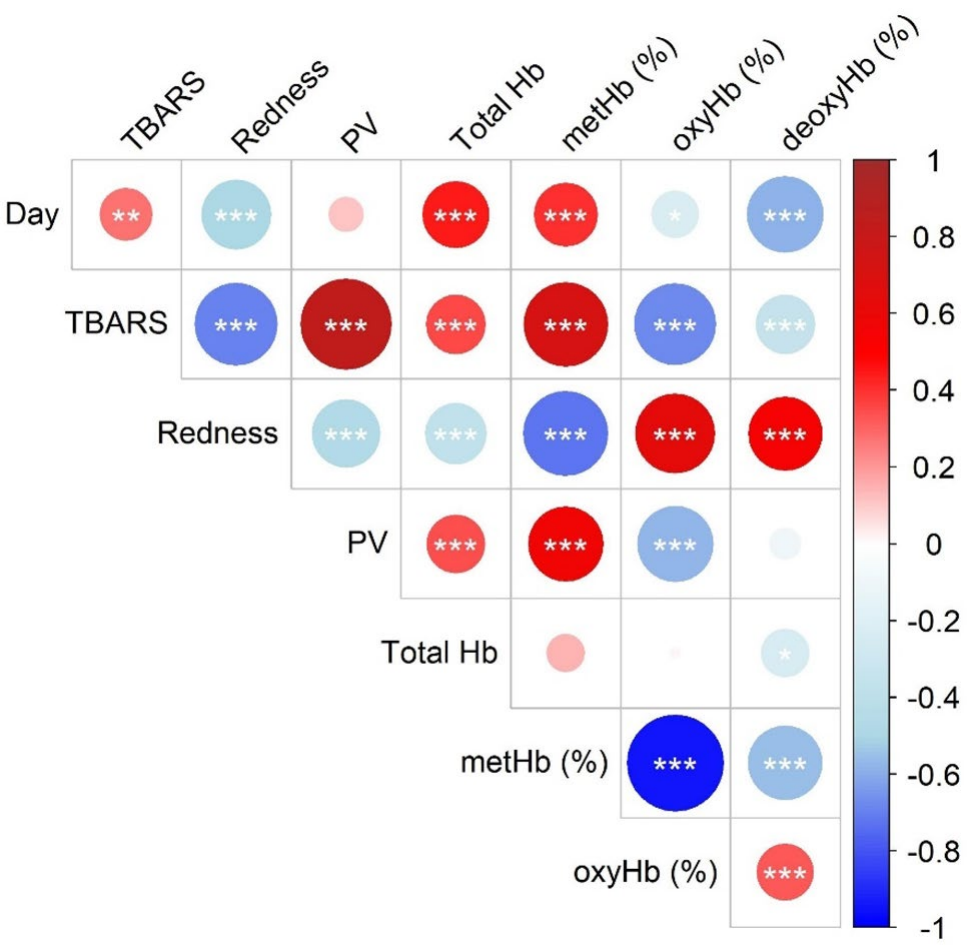
Spearman correlation between measures of lipid oxidation and storage time. Significant correlations are denoted with asterisks as *p* < 0.001 = ***, *p* < 0.01 = **, and *p* < 0.05 = *. The red color indicates a positive correlation, and the blue color denotes a negative correlation.

Spearman’s correlation analyses were conducted to investigate the relationship between all the studied factors (Fig. 5): days of storage, TBARS, redness (i.e., a*-value), PV, total soluble Hb, and soluble metHb, oxyHb, and deoxyHb in all variants of WCM samples (i.e., at different pH and RBC). A strong negative correlation (r_s_ = − 0.75; *p* = $1.03\times {10}^{-12}$) was found between TBARS and redness across all WCM samples, including those with different ratios between intact/lysed RBCs and varying pH. This reinforces the strong link between formation of the brown/grey metHb form, and lipid oxidation, and was in agreement with our previous study where we suggested a*-value analyses as a potential indirect tool to determine lipid oxidation in Hb-fortified WCM systems^32^. Supporting this link, a strong negative correlation was also found between redness and the relative level of soluble metHb (i.e., % of the total Hb) (Supplementary, Fig. 1–2) (r_s_ = − 0.80; *p* = 3$.39\times {10}^{-15}$) and a strong positive correlation was found between relative levels of soluble metHb and TBARS (r_s_ = 0.81; *p* = 9$.67\times {10}^{-16}$). When using *absolute* metHb-levels in the calculations (Figs. 1G and 2I), only intermediate positive correlations were found to redness (r_s_ = 0.62; *p* = 1.$18\times {10}^{-7}$) and TBARS (r_s_ = 0.58; *p* = 9.$51\times {10}^{-7}$), probably due to the interference from binding of Hb to WCM. The significant correlations (*p* < 0.05) between %metHb, redness and TBARS are in line with the explained mechanisms behind Hb-mediated oxidation, i.e., efficient cleavage of hydroperoxides by metHb or its released hemin. No significant correlation was found between TBARS and the concentration of total soluble Hb, which could be explained by the triple phenomena occurring in a muscle system with RBCs; hemolysis, heme/Hb-binding and porphyrin ring destruction.

## Conclusion

WCM samples that initially contained lysed RBCs (25–100%), translating to 17.5–71 µM soluble Hb, were subjected to rapid lipid oxidation during cold storage while starting the storage with only intact RBCs prolonged the lag-phase by 1 day. This reveals that if hemolysis can be delayed industrially, processors of blood-rich fish raw materials can obtain enough time e.g., for short transports or holdings prior to further valorization. However, even when starting the storage with 100% intact RBCs, lipid oxidation generally increased at pH < 7.2 when only 7.1 µM Hb was measured in the supernatant; stressing the highly pro-oxidative ability of fish Hbs, particularly in the metHb form. To mitigate this reaction, components of plasma can clearly play a profound role, already at plasma levels of 3% (v/w), which could be explored further when searching for natural antioxidants to stabilize fish. Significant suppression (*p* < 0.05) of Hb-mediated oxidation was also observed during the 7-day storage trial when increasing pH of the WCM-RBC model from the typical post-mortem pH 6.4–6.8, up to 7.2–7.6.

Altogether, it was not possible to keep RBCs fully intact for an extended amount of time under the conditions tested. Yet, our study could prove that minimizing initial lysis delays the onset of membrane lipid oxidation. To successfully use RBC-integrity as an antioxidant method, complete integrity should however be aimed for since very low lysis levels were sufficient to initiate oxidation. Thus, to avoid strongly lysing conditions such as osmotic or mechanic-stress would clearly be advantageous during fish processing, but parallel subjection to antioxidants and/or pH-elevations should be carried out, preferably early. In this way, currently underutilized blood rich fish raw materials such as side-streams from filleting or small pelagic fish species could be maintained in the food chain to a better extent, contributing to a more sustainable seafood production system.

### Supplementary Information

Supplementary Information.

## Data Availability

The datasets used and analyzed during the current study are available from the corresponding author on reasonable request.
