# Supplementary material for: Relationship between hemolysis and lipid oxidation in red blood cell-spiked fish muscle; dependance on pH and blood plasma
Source: Sci Rep. 2024 Jan 23;14:1943. doi: 10.1038/s41598-024-52090-8 (PMC10803305; doi:10.1038/s41598-024-52090-8)
Supplement: Supplementary file 1 — Supplementary Information. [file 41598_2024_52090_MOESM1_ESM.docx]

**Supplementary Information**

Title: Relationship between hemolysis and lipid oxidation in red blood cell-spiked fish muscle; dependance on pH and blood plasma

Semhar Ghirmai*^a^, Annika Krona^b^, Haizhou Wu^a^, James Whalin^a^, Michael Axelsson^c^ and Ingrid Undeland^a^

**Author names:**

*Corresponding author

*Semhar Ghirmai*^a^*

Email address: semhar_ghirmai@hotmail.com

Tel. no.: +46739082457

*Annika Krona^b^*

Email address: [annika.krona@ri.se](mailto:annika.krona@ri.se)

Tel. no.: +46 10 516 66 38

*Haizhou Wu^a^*

Email address: [haizhou@chalmers.se](mailto:haizhou@chalmers.se); haizhou@mail.hzau.edu.cn

*James Whalin*

Email address: whalin@chalmers.se

*Michael Axelsson^c^*

Email address: [Michael.axelsson@bioenv.gu.se](mailto:Michael.axelsson@bioenv.gu.se)

*Ingrid Undeland^a^*

Email address: [undeland@chalmers.se](mailto:undeland@chalmers.se)

**Affiliations:**

^a^Chalmers University of Technology, Department of Life Sciences, Division of Food and Nutrition Science SE-412 96 Gothenburg, Sweden

^b^RISE Research Institutes of Sweden, Department Agriculture and Food, Division Bioeconomy and Health, Frans Perssons väg 6, SE-402 29 Gothenburg, Sweden

^c^Gothenburg University, Department of Biological and Environmental Sciences, Medicinaregatan 18a, SE-413 90 Gothenburg, Sweden

**6**

**Fig. 2.** The Hb form of the supernatant from the RBC spiked WCM system at pH ranging from pH 6.4 up to 7.6, with or without addition of plasma. Panel A-C show the percentages of oxy-, deoxy- and metHb, respectively. Data is shown as mean ± SD (n=2).


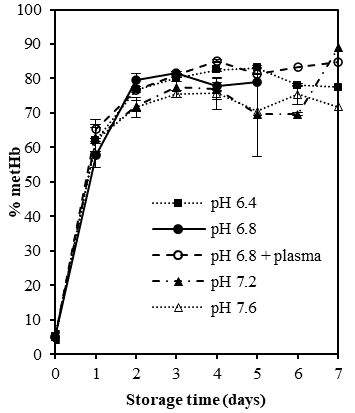


**C**


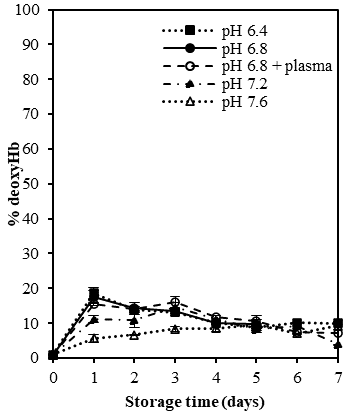


**B**


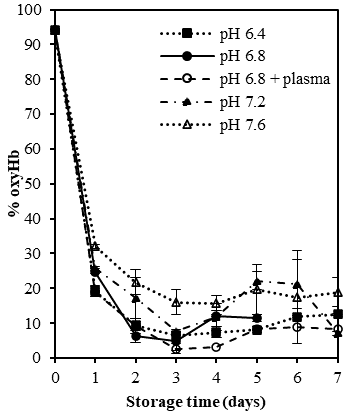


**A**

**Fig. 1.** The Hb form of the supernatant from the WCM system spiked with intact or lysed RBCs. Panel A-C visualize, oxy-, deoxy- and metHb percentages, respectively. Data is shown as mean ± SD (n=2).


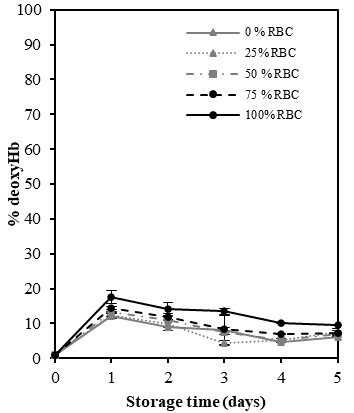


**B**


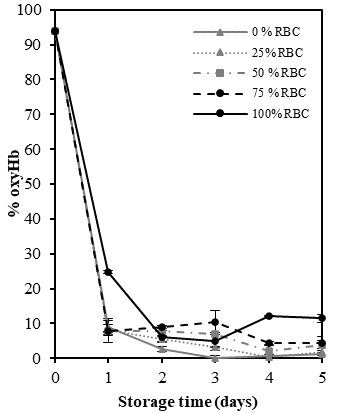


**A**


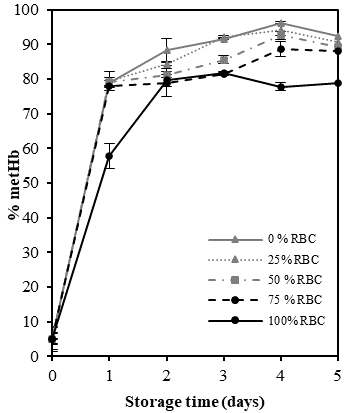


**C**

**Fig. 3.** Development of PV (panel A) and TBARS (panel B) for WCM sample spiked with fully lysed RBCs (0% intact RBCs) or 50 % intact RBCs + 50 % lysed RBCs.


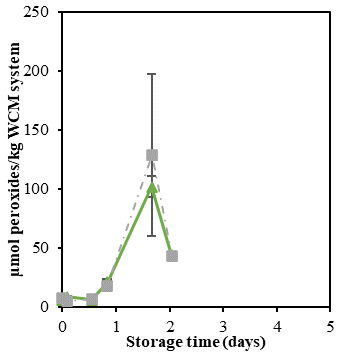


**A**


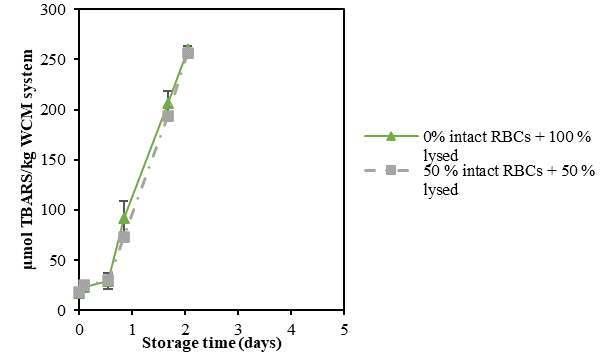


**B**


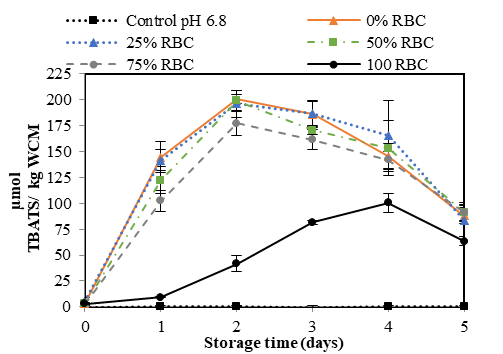


**B**


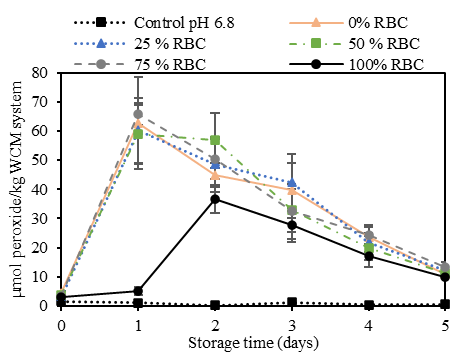


**A**

**Fig. 4.** Development of PV (panel A) and TBARS (panel B) for WCM with addition of 0, 25, 75 or 100 % intact RBCs.


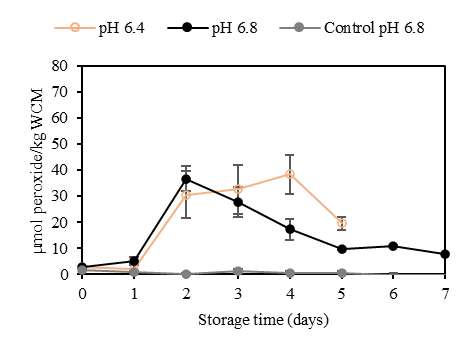


**A**


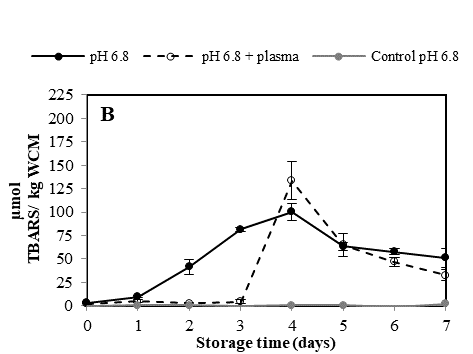


**Fig. 5.** Development of PV (panel A) and TBARS (panel B) for WCM with addition intact RBCs with and without 3% plasma.


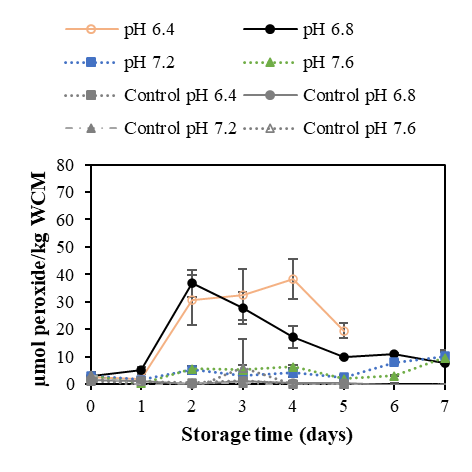

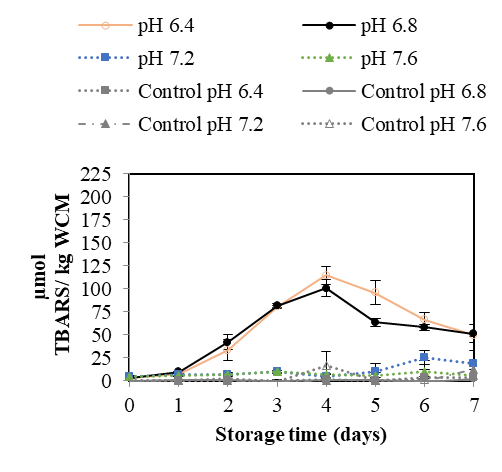


**Fig. 6.** Development of PV (panel A) and TBARS (panel B) of model systems with RBCs added to WCMs with various pH.

| ANOVA |  |  | Mixed model |  |  |
| --- | --- | --- | --- | --- | --- |
| Trt | Significance | LSMean | Trt | Significance | LSMean |
| 25% RBC pH 6.8 | A | 1,916667 | 25% RBC pH 6.8 | A | 1,916667 |
| 0% RBC pH 6.8 | A | 1,9 | 0% RBC pH 6.8 | A | 1,9 |
| 50% RBC pH 6.8 | A | 1,866667 | 50% RBC pH 6.8 | A | 1,866667 |
| 75% RBC pH 6.8 | AB | 1,85 | 75% RBC pH 6.8 | A | 1,85 |
| 100% RBC pH 6.8 | B | 1,475 | 100% RBC pH 6.8 | B | 1,475 |
| Control | C | -0,21667 | Control | C | -0,21667 |

**Fig. 7.** Difference in statistical output for log transformed TBARS of lysed RBC experiment. Mixed model accounts for repeated measures whereas the ANOVA does not. Main conclusion of the 100% RBC causing less lipid oxidation is unchanged.


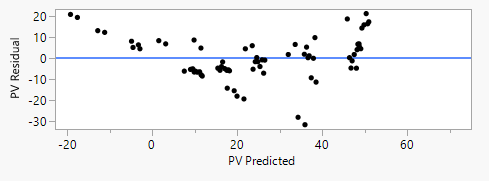

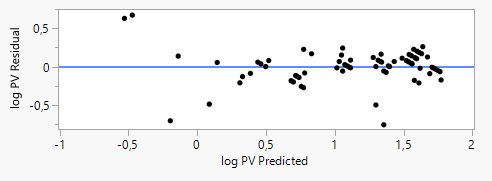


**Fig. 8.** Comparison of residual plots for original data (Lysed RBC PV; panel A) and log10 transformed data (Lysed RBC PV; Panel B)
